# Parent soil type modulates biochar and mowing effects on soil microbial communities in karst region

**DOI:** 10.3389/fmicb.2025.1680847

**Published:** 2025-10-28

**Authors:** Xun Yi, Wang–Lan Tao, Han–Han Zhou, Shi–Wen Zhu, Xin–Yue Wang, Zhi–Lin Dong, Si–Yu Gao, Xian–Kun Li, Xu–Xin Song

**Affiliations:** ^1^College of Tourism and Landscape Architecture, Guilin University of Science and Technology, Guilin, China; ^2^College of Plant and Ecological Engineering, Guilin University of Technology, Guilin, China; ^3^Guangxi Key Laboratory of Plant Conservation and Restoration Ecology in Karst Terrain, Guangxi Institute of Botany, Guangxi Zhuang Autonomous Region and Chinese Academy of Sciences, Guilin, China; ^4^Nonggang Karst Ecosystem Observation and Research Station of Guangxi, Chongzuo, China

**Keywords:** calcareous soil, microbial diversity, microbial abundance, red soil, soil properties

## Abstract

**Introduction:**

Karst ecosystems are highly susceptible to degradation due to their inherent fragility and poor soil conditions. Soil microorganisms play a crucial role in nutrient cycling and ecosystem recovery. While biochar application has been shown to enhance microbial activity, its interaction with mowing–a common grassland management practice-and whether such effects vary with soil type remain unclear.

**Methods:**

A 1-year mesocosm experiment was conducted using red and calcareous soils from southwest China, with four treatments: control (CK), biochar (B), mowing (M), and combined biochar–mowing (BM). Highthroughput sequencing was used to assess microbial abundance, alpha diversity, and community structure.

**Results:**

We found that the individual and combined effects of biochar and mowing on soil microbial communities differed significantly between soil types. Biochar-only treatment consistently increased bacterial and fungal abundance and richness in both soil types. However, significant increases in fungal diversity, evenness and bacterial simpson were observed only in red soil. Mowing enhanced microbial abundance, richness, and diversity in red soil but had no significant effect in calcareous soil. The highest microbial abundance and richness under the combined BM treatment in red soil suggest a potential synergistic effect between biochar and mowing. Biochar significantly increased the relative abundances of dominant bacterial *phyla-Proteobacteria* and *Actinobacteria*–while decreasing *Chloroflexi* in red soil, with minimal changes observed in calcareous soil. Similarly, it elevated the relative abundances of fungal phyla *Ascomycota* and *Basidiomycota*, but reduced Chytridiomyota in red soil, whereas calcareous soil showed less pronounced shifts. Strong correlations were observed between soil properties and microbial community structure, particularly in red soil.

**Conclusion:**

These findings indicate that biochar and mowing can jointly improve soil microbial communities, offering potential for restoring degraded karst grasslands. However, their effectiveness is strongly mediated by parent soil type.

## 1 Introduction

Karst ecosystems are prone to severe degradation (rocky desertification), due to their fragile geological structure and hydrological cycles (Gutiérrez et al., 2014), leading to reduced soil productivity, biodiversity loss, weakened carbon sequestration, and diminished ecosystem services, with significant environmental and socioeconomic impacts (Jiang et al., 2014). Consequently, ecological restoration measures such as forage cultivation and soil amendment are commonly adopted for mitigation (Zhou et al., 2020; Zhang et al., 2021). Biochar is widely utilized as a soil amendment for karst due to its porous structure and surface functional groups (Guo et al., 2024). Besides, mowing represents a prevalent management practice during forage cultivation system (Blüthgen et al., 2012), yet its individual and interactive effects with biochar on soil properties and biota in karst ecosystems remain poorly constrained. Furthermore, given the critical role of soil microorganisms in sustaining ecological functions (Bardgett and van der Putten, 2014), their acute sensitivity to environmental changes makes community structure, composition, and diversity vital indicators of soil health (Fierer et al., 2021). Consequently, there is growing scientific focus on whether and how anthropogenic management practices (e.g., biochar application and mowing) influence soil microbial community structure (Thakur and Geisen, 2019).

It is noteworthy that both biochar application and mowing management can alter soil-related variables, thereby modifying microbial diversity, abundance, and composition (Li et al., 2023). Biochar possesses a high aromatic carbon content, extensive porosity, and substantial surface area, which collectively improve soil aeration, water-holding capacity, and nutrient retention. Simultaneously, it influences microbial communities through mechanisms including provision of microbial habitats, adsorption of toxic substances, and modulation of soil pH and nutrient status (Lehmann et al., 2011). Studies indicate that biochar amendment significantly restructures soil bacterial and fungal community composition while enhancing the relative abundance of dominant microbial taxa (Lei et al., 2023). Mowing is a non-destructive practice that removes surface vegetation, thereby reducing root exudate production and altering litter quantity, which leads to a decline in soil organic matter input (Gilmullina et al., 2020). Concurrently, the reduction in vegetation coverage modifies light penetration, resulting in increased soil evaporation that affects soil temperature and moisture content. These alterations further shift aboveground-belowground carbon allocation patterns (Miao et al., 2020). Consequently, mowing induces structural shifts in soil microbial communities: bacterial communities remain largely unaffected, whereas a significant increase in fungal relative abundance is consistently documented (Li et al., 2020).

Given that both interventions alter the composition and diversity of soil microbial, and considering the crucial role of microbial diversity in maintaining soil functions stability, increasing microbial diversity has become a key strategy for rehabilitating degraded ecosystems (Delgado-Baquerizo et al., 2016). Divergent effects of biochar and mowing on soil microbial communities, in both magnitude and direction, likely arise from variations in background soil conditions (Wang et al., 2025). In acidic soils, biochar application can neutralize soil acidity and provide a stable carbon (Arwenyo et al., 2023), alleviate aluminum toxicity, and thereby likely enhance microbial activity to improve the overall health of acidic soils by promoting microbial metabolic processes (Xia et al., 2023). Conversely, in alkaline soils that naturally contain higher nutrient levels, the effectiveness of biochar may be markedly diminished, with its influence on soil microbial communities being relatively minimal (Zhou et al., 2019). However, the effects of mowing were not directly mediated by soil pH but rather indirectly through alterations in soil moisture, and nutrient availability, which subsequently enhanced specific microbial diversity indices by modifying microbial biomass. It may also be subject to the influences of aboveground biomass and diversity, which could potentially lead to variations in microbial biomass (Song et al., 2020).

The soil types in this karst area are mainly red and calcareous soils, both of which are facing serious degradation problems (Zhong et al., 2022). Red soil is the product of intense biological enrichment and subsequent desilication–aluminization weathering processes under the humid subtropical bioclimatic conditions, characterized by low pH, heavy texture, and high exchangeable ion content (Yang et al., 2017). In contrast, calcareous soil develops on carbonate rock weathering materials in tropical and subtropical regions, featuring neutral pH, strong weathering and leaching effects, and low degrees of calcium depletion (Yan et al., 2022). We report the results of a 1–year mesocosm experiment investigating the effects of biochar application, mowing, and their interactions on soil microbial diversity, abundance, composition, and soil nutrient status across different soil parent materials in a degraded karst region, aiming to enhance soil nutrient availability and improve microbial community structure. We hypothesized that (1) the application of biochar and mowing in karst grassland would change the soil microbial diversity, abundance and composition; (2) different types of parent soil respond differently to relevant management measures and have a certain degree of soil dependence; (3) changes in some soil variables can lead to alterations in microbial diversity, community structure and composition.

## 2 Materials and methods

### 2.1 Study site

The experiment was conducted in a greenhouse in Guilin City, located in the karst region of southwest China’s Guangxi Zhuang Autonomous Region (coordinates: 24°15′2′′–26°23′30′′N, 109°36′5′′–111°29′3′′ E). This region experiences a subtropical monsoon climate, with an average annual temperature of approximately 19.1 °C. The lowest monthly mean temperature occurs in January (8 °C), while the highest occurs in July (28 °C). Mean annual precipitation ranges from 1160 to 1378 mm, with a distinct seasonal pattern. The study area is mountainous, interspersed with both karst and non-karst regions. Two parent material soil types were used: iron–rich red soil (pH 5.2–6.6) and calcareous soil (pH 7.8–8.2), which were collected as topsoil from nearby karst grasslands, respectively. The soils were air-dried and homogenized in preparation for potting experiments.

### 2.2 Experimental design and sample collection

We designed a mesocosm experiment to investigate the effects of biochar application and mowing on the soil microbial community structure in two soil types of karst grasslands on March 1, 2022. The experiment was conducted as a randomized block design, with four blocks (replicates) (*n* = 4) arranged across the greenhouse space. Each block contained all treatment combinations (2 soil types × 2 biochar levels × 2 mowing regimes = 8 pots per block). Within each block, pot positions were randomly assigned using a random number generator in Excel to ensure unbiased treatment distribution. To minimize the influence of environmental gradients–such as light intensity, temperature, and humidity–blocks were arranged along both east–west and north–south axes, which are known to affect microclimatic conditions in greenhouse settings. Additionally, all pots were rotated weekly within their respective blocks to further homogenize exposure to light and temperature and reduce positional bias.

A total of 32 pots were used, containing two types of soil. The initial physicochemical properties of the soils prior to the experiment are presented in Supplementary Table 1. Each pot has an upper diameter of 30 cm, a bottom diameter of 23 cm, and a height of 23 cm, yielding a volume of approximately 8364 cm^3^. Each pot was filled with about 8 kg of soil. Biochar was produced by pyrolyzing corn stalks at 500 °C and applied to the soil at a rate of 10% (w/w; 100 g kg^−1^) prior to planting. This corresponded to an average of 0.8 kg of biochar per pot, based on the dry soil weight. The biochar charaterization are presented in Supplementary Table 2. Each pot was then planted with 20 tall fescue plants (*Festuca arundinacea* Scherb.), a C_4_grass adapted to the climate of Guilin. The plants were watered daily to maintain soil moisture at 70%–80% of the water-holding capacity. Regular manual weeding was carried out to control weeds, prevent resource competition, and maintain consistent experimental conditions. No fertilization or liming was applied to the potted plants in order to isolate the effects of biochar as the controlled variable.

On March 15, 2023 (1–year post–planting), we carried out the mowing treatment, prior to the treatment, foliar height was measured and allometric equations was employed to estimate height–biomass relationships (Chave et al., 2014). The allometric equations are as follow:

*B* = 0.0993 × *H*^0.9274^ (R^2^ = 0.6628)

Among them:

*H = Plant height*

*B = Aboveground biomass*

To achieve 50% removal of aboveground biomass, specific height–based excisions were performed on plants. Mean canopy heights were recorded as 10 cm (non–biochar) versus 20 cm (biochar–amended) in red soils, and 18 cm (non–biochar) versus 25 cm (biochar–amended) in Calcareous soils. Excised plant material was systematically removed from experimental plots.

Full harvest of residual plant material occurred on March 30, 2023, followed by desiccation at 65 °C for 48 h with subsequent quantification of aboveground and belowground biomass. Soil cores (0–15 cm depth) were collected from each pot and sieved (2 mm mesh) to eliminate rock fragments and root residues. The soil samples were bifurcated into two subsamples: Air-dried specimens were homogenized through 0.15 mm mesh sieving; Field–moist specimens were immediately transported to laboratory facilities under refrigerated preservation (4 °C). Prior to analysis, field-moist soils were re-sieved (2 mm mesh) and homogenized through thorough mixing. Air-dried soil specimens were subjected to quantitative analyses for pH, soil organic carbon (SOC), total nitrogen (TN), total phosphorus (TP), cation exchange capacity (CEC), exchangeable calcium (ECa), and exchangeable magnesium (EMg). Concurrently, field-moist soil samples were employed for the determination of microbial biomass carbon (MBC), ammonium (NH_4_^+^), and nitrate (NO_3_^–^) concentrations, with analyses conducted immediately following sample collection to preserve biochemical integrity.

### 2.3 Chemical analysis of the soil samples

The physicochemical properties of the soil samples were analyzed through standardized laboratory procedures. Soil pH was determined via potentiometric analysis using a calibrated pH meter. Gravimetric analysis was performed to quantify soil water content (SWC) by measuring mass loss after oven-drying at 105 °C to constant weight. Soil organic carbon (SOC) content was analyzed using the potassium dichromate oxidation method with external heating in concentrated sulfuric acid medium. Total nitrogen (TN) concentrations were determined through sulfuric acid-catalyzed digestion followed by automated flow injection analysis (AA3 system). For total phosphorus (TP) measurement, samples underwent sulfuric-perchloric acid digestion and subsequent quantification using molybdenum-antimony anti-spectrophotometry. Microbial biomass carbon (MBC) was assessed through chloroform fumigation-extraction methodology with K_2_SO_4_ solution. The available N (NH_4_^+^ and NO_3_^–^) were extracted using KCl solution and analyzed by continuous flow analytical techniques. Cation exchange capacity (CEC) was determined through ammonium acetate saturation followed by flame photometric detection of displaced cations. Complexed iron (CoFe) fractions were extracted using sodium pyrophosphate solution and quantified spectrophotometrically. Exchangeable base cations (Ca_2_^+^ and Mg_2_^+^) were determined through sequential extraction with ammonium acetate followed by atomic absorption spectroscopic analysis.

### 2.4 Microbial high-throughput sequencing

Microbial community analysis was conducted using the following standardized high–throughput sequencing pipeline: Bacterial 16S rRNA gene fragments were amplified using primers 338F (5′–ACTCCTACGGGAGGCAGCAG–3′) and 806R (5′–GGACTACHVGGGTWTCTAAT–3′), while fungal ITS regions were amplified with primers ITS1F (5′–CTTGGTCATTTAGAGGAAGTAA–3′) and ITS2R (5′–GCTGCGTTCTTCATCGATGC–3′). Sequencing was performed by Shanghai Majorbio Biomedical Technology Co., Ltd. on an Illumina NovaSeq platform. Sequencing depth is 50,000 reads per sample; the dilution threshold is 40,000 reads per sample. Raw paired-end reads were first quality-controlled using fastp (v0.19.6). Overlapping reads were then assembled using FLASH (v1.2.11). Quality-filtered sequences were clustered into Operational Taxonomic Units (OTUs) at a 97% similarity threshold via UPARSE (v11), with chimeras removed using the UCHIME algorithm. To mitigate sequencing depth bias in α - diversity analyses, all samples were rarefied, maintaining an average Good’s coverage of 99.09%. Taxonomic annotation of OTUs was performed against the SILVA 16S rRNA database (v138) using the RDP Classifier (v2.13) with a 70% confidence threshold, enabling hierarchical compositional profiling from phylum to genus levels. The data presented in the study are deposited in the NCBI Sequence Read Archive (SRA) repository, accession number PRJNA1338870.

### 2.5 Statistical analysis

Microbial α–diversity was calculated using mother (version v.1.30.1). This includes richness indices (Chao1, ACE), diversity indices (Shannon, Simpson), and evenness index (Pielou).

Chao1 Index Formula:


$$\begin{array}{c} S_{c\,h\,a\,o\,1}=S_{o\,b\,s}+\frac{n_{1}\,\left(n_{1}-1\right)}{2\,\left(n_{2}+1\right)} \end{array}$$

Among them:

*S*_*chao*1_ = The estimated number of OTUs

*S*_*obs*_ = The actual number of OTUs observed

*n*_1_ = The number of OTUs containing only one sequence (such as “singletons”)

*n*_2_ = The number of OTUs containing only two sequences (such as “doubletons”)

ACE Index Formula:


$$<0.80\,S_{a\,b\,u\,n\,d}+\frac{S_{r\,a\,r\,e}}{C_{A\,C\,E}}+\frac{n_{1}}{C_{A\,C\,E}}\,{\tilde{\gamma }}_{A\,C\,E}^{2},f\,o\,r\,{\hat{\gamma }}_{A\,C\,E}\geq 0.80$$

Among them:


$$\begin{array}{cc} N_{r\,a\,r\,e}=\sum_{i=1}^{a\,b\,u\,n\,d}, & C_{A\,C\,E}=1-\frac{n_{1}}{N_{r\,a\,r\,e}} \end{array}$$

$${\hat{\gamma }}_{A\,C\,E}^{2}=m\,a\,x\,\left\lfloor \frac{S_{r\,a\,r\,e}}{C_{A\,C\,E}}\,\frac{\sum_{i=1}^{a\,b\,u\,n\,d}i\,\left(i-1\right)\,n_{i}}{N_{r\,a\,r\,e}\,\left(N_{r\,a\,r\,e}-1\right)}-1,0\right\rfloor$$

$${\tilde{\gamma }}_{A\,C\,E}^{2}=m\,a\,x\,\left[{\hat{\gamma }}_{A\,C\,E}^{2}\,\left\{1+\frac{N_{r\,a\,r\,e}\,\left(1-C_{A\,C\,E}\right)\,\sum_{i=1}^{a\,b\,u\,n\,d}i\,\left(i-1\right)\,n_{i}}{N_{r\,a\,r\,e}\,\left(N_{r\,a\,r\,e}-C_{A\,C\,E}\right)}\right\},0\right]$$

*n*_*i*_ = The number of OTUs containing a sequence

*S*_*rare*_ = The number of OTUs containing “abund” sequences or less than “abund”

*S*_*abund*_ = More than the number of OTUs in the “abund” sequence

abund = The threshold for “Advantage “OTU is set to 10 by default

Shannon Index Formula:


$$H_{s\,h\,a\,n\,n\,o\,n}=-\overset{S_{o\,b\,s}}{\underset{i=1}{\sum }}\frac{n_{i}}{N}\,l\,n\,\frac{n_{i}}{N}$$

Among them:

*S*_*obs*_ = The actual number of OTUs observed

*n*_*i*_ = The number of sequences contained in the i-th OTU

*N* = All the sequence numbers

Simpson Index Formula:


$$1-D_{s\,i\,m\,p\,o\,n\,s}=1-\frac{\sum_{i=1}^{S_{o\,b\,s}}n_{i}\,\left(n_{i}-1\right)}{N\,\left(N-1\right)}$$

Among them:

*S*_*obs*_ = The actual number of OTUs observed

*n*_*i*_ = The number of sequences contained in the i-th OTU

*N* = All the sequence numbers

Pielou Index Formula:


$$J=\frac{H}{l\,n\,\left(S\right)}$$

Among them:

*H* = Shannon Index

*S* = The number of species in the ecological community(sobs)

*ln* = The logarithm to the base “e”

A three-way analysis of variance (ANOVA) was conducted using a general linear model (GLM) to examine the effects of soil type, biochar application, and mowing on soil microbial α-diversity indices (OTUs, Chao1, ACE, Shannon, Simpson and Pielou). Soil type, biochar, and mowing were treated as fixed effects, while block was included as a random effect to account for spatial heterogeneity. A significant interaction indicates that the effects of biochar and/or mowing on microbial communities depend on soil type. To further investigate whether soil type modulates the impacts of biochar and mowing across different microbial community metrics, two-way ANOVAs were performed separately for each soil type. Tukey’s post hoc tests were used to identify significant differences among treatment combinations within each soil type. Each treatment combination was replicated four times (*n* = 4), with one pot per replicate. All analyses were conducted in SPSS (Version 27.0), with statistical significance set at *p* < 0.05. The composition of microbial communities, redundancy analysis (RDA), and Pearson correlation analysis were conducted using relevant packages in R (version 4.5.0) and Origin (version 2024), with significance assessed at *p* < 0.05. All data are presented as mean ± standard error (SE), unless otherwise specified. The assumptions of normality and homogeneity of variances were evaluated using residual plots; data were log-transformed when necessary to meet model assumptions.

## 3 Results

### 3.1 Microbial α diversity

Three-way ANOVA indicated that soil type (ST), biochar application (B), and mowing management (M) significantly influenced the diversity index of bacterial and fungal communities (Table 1). Among them, soil type was the most significant driving factor, exerting a highly significant impact on all measured diversity indices, including OTUs, Chao1, ACE, Shannon, Simpson, and Pielou (*p* < 0.001). Biochar significantly affected OTUs, Chao1, and ACE of both bacteria and fungi (*p* < 0.001). Mowing significantly influenced the richness of bacteria (OTUs: *p* < 0.01; Chao1: *p* = 0.001; ACE: *p* < 0.01) and strongly affected fungal diversity and evenness (Shannon: *p* < 0.001; Simpson: *p* < 0.001; Pielou: *p* < 0.001), with a marginally significant effect on bacterial Shannon index (*p* = 0.054). Significant two-way interactions included: ST × B affecting the simpson of both bacterial and fungal communities and fungal richness (*p* < 0.05), ST × M significantly affecting all diversity indices of both communities (*p* < 0.01), and B × M significantly shaping bacterial richness (OTUs, Chao1, ACE; *p* < 0.05) and fungal diversity (Shannon: *p* = 0.001; Simpson: *p* = 0.005; Pielou: *p* < 0.001). Notably, the three-way interaction (ST × B × M) was significant only in terms of fungal diversity and evenness (Shannon: *p* = 0.004; Simpson: *p* = 0.006; Pielou: *p* < 0.001).

**TABLE 1:** Summary of three-way ANOVA analysing the effects of soil types (ST), biochar (B), mowing (M) and their interactive effects on soil microbial OTUs, Chao 1, ACE, Shannon, Simpson and Peilou, and using ST, B, M and their interaction as fixed terms.

Microbial	OTUs		Chao1		ACE		Shannon		Simpson		Pielou	
	*F*	*P*	*F*	*P*	*F*	*P*	*F*	*P*	*F*	*P*	*F*	*P*
**Bacteria**												
ST	731.618	<0.001^**^	1025.38	<0.001^***^	1011.182	<0.001^***^	277.083	<0.001^***^	143.909	<0.001^***^	142.96	<0.001^***^
B	39.368	<0.001^***^	63.81	<0.001^***^	67.087	<0.001^***^	2.615	0.121	7.107	0.014^*^	0.067	0.798
M	8.878	0.007^**^	15.091	0.001^**^	12.09	0.002^**^	4.16	0.054	2.558	0.125	2.128	0.159
B * M	5.437	0.03^*^	6.016	0.023^*^	6.049	0.023^*^	0.452	0.509	0.64	0.433	0.002	0.968
ST * B	0.119	0.734	1.27	0.273	1.62	0.217	1.445	0.243	13.929	0.001^**^	4.818	0.04^*^
ST * M	11.294	0.003^**^	13.386	0.001^**^	12.105	0.002^**^	8.455	0.008^**^	3.482	0.076	5.563	0.028^*^
ST * B ^*^ M	0.09	0.767	0.014	0.907	0.174	0.681	0.211	0.65	0.071	0.792	0.067	0.798
**Fungi**												
ST	120.832	<0.001^***^	115.281	<0.001^***^	113.291	<0.001^***^	944.752	<0.001^***^	71.604	<0.001^***^	835.567	<0.001^***^
B	35.797	<0.001^***^	44.371	<0.001^***^	42.517	<0.001^***^	0.531	0.474	4.678	0.042^*^	9.633	0.005^**^
M	0.041	0.841	0.037	0.849	0.012	0.916	56.066	<0.001^***^	17.901	<0.001^***^	79.342	<0.001^***^
B* M	0.207	0.654	0.255	0.619	0.196	0.663	16.651	0.001^**^	9.892	0.005^**^	21.571	<0.001^***^
ST * B	11.045	0.003^**^	8.337	0.009^**^	8.706	0.008^**^	0.01	0.922	4.943	0.037^*^	0.222	0.642
ST * M	3.161	0.09	3.222	0.087	3.245	0.086	27.324	<0.001^***^	15.44	0.001^**^	35.53	<0.001^***^
ST * B * M	0.8	0.381	0.965	0.337	0.926	0.347	10.435	0.004^**^	9.411	0.006^**^	19.012	<0.001^***^

Design variables were taken as random-effects terms. An asterisk (*) indicates significant differences (*p < 0.05;

**p < 0.01;

***p < 0.001).

The two-factor variance analysis conducted within each soil type revealed soil-specific responses to management measures (Table 2). In red soil, biochar significantly affected bacterial OTUs (*p* < 0.01), Chao1 (*p* < 0.001), ACE (*p* < 0.001), and Simpson (*p* < 0.01), as well as fungal OTUs, Chao1, and ACE (*p* < 0.001). Mowing significantly enhanced bacterial richness (OTUs, Chao1, ACE; *p* < 0.01) and diversity (Shannon: *p* < 0.05), and significantly improved fungal all measured diversity indices, including OTUs, Chao1, ACE, Shannon, Simpson, and Pielou (*p* < 0.001). The B × M interaction was significant for fungal Shannon (*p* < 0.01), Simpson (*p* < 0.05), and Pielou (*p* = 0.001). In contrast, in calcareous soil, biochar significantly increased bacterial OTUs (*p* = 0.001), Chao1 (*p* = 0.001) ACE (*p* = 0.001), and shannon (*p* < 0.05), and improved fungal OTUs (*p* = 0.001), Chao1 (*p* = 0.001), ACE (*p* = 0.001) and evenness (Pielou: *p* < 0.01). Mowing had no significant effect on all bacterial indicators, with only marginally significant improvement in fungal Shannon (*p* < 0.05).

**TABLE 2:** Summary of two-way ANOVA analysing the effects of biochar (B), mowing (M) and their interactive effects on soil microbial OTUs, Chao 1, ACE, Shannon, Simpson and Peilou in red soil (RS) and calcareous soil (CS), and using T, N, G and their interaction as fixed terms.

Microbial		OTUs		Chao1		ACE		Shannon		simpson		Pielou	
		*F*	*P*	*F*	*P*	*F*	*P*	*F*	*P*	*F*	*P*	*F*	*P*
**Bacteria**													
RS	B	17.062	0.003^**^	33	<0.001^***^	42.058	<0.001^***^	0.068	0.8	12.314	0.007^**^	2.524	0.147
	M	19.506	0.002^**^	22.601	0.001^**^	22.726	0.001^**^	9.693	0.012^*^	3.613	0.09	6.107	0.035^*^
	B*M	2.002	0.191	2.165	0.175	1.958	0.195	0.507	0.494	0.342	0.573	0.02	0.891
CS	B	20.57	0.001^**^	27.718	0.001^**^	22.03	0.001^**^	7.466	0.023*	1.788	0.214	3.604	0.09
	M	0.068	0.8	0.03	0.866	0	0.999	0.708	0.422	0.354	0.567	0.731	0.415
	B*M	3.255	0.105	3.891	0.08	3.81	0.083	0.045	0.836	0.28	0.61	0.1	0.759
**Fungi**													
RS	B	111.017	<0.001^***^	245.47	<0.001^***^	180.488	<0.001^***^	0.259	0.623	4.777	0.057	2.127	0.179
	M	61.598	<0.001^***^	68.113	<0.001^***^	51.601	<0.001^***^	61.084	<0.001^***^	16.536	0.003^**^	67.851	<0.001^***^
	B*M	3.035	0.115	3.921	0.079	3.823	0.082	20.195	0.002^**^	9.585	0.013^*^	24.887	0.001^**^
CS	B	21.664	0.001^**^	22.74	0.001^**^	22.396	0.001^**^	0.449	0.52	0.199	0.666	11.891	0.007^**^
	M	0.621	0.451	0.64	0.444	0.717	0.419	5.758	0.04^*^	4.36	0.066	8.021	0.02^*^
	B*M	0.457	0.516	0.552	0.476	0.493	0.5	0.811	0.391	0.175	0.685	0.071	0.796

Design variables were taken as random-effects terms. An asterisk (*) indicates significant differences (*p < 0.05;

**p < 0.01;

***p < 0.001).

**FIGURE 1:**
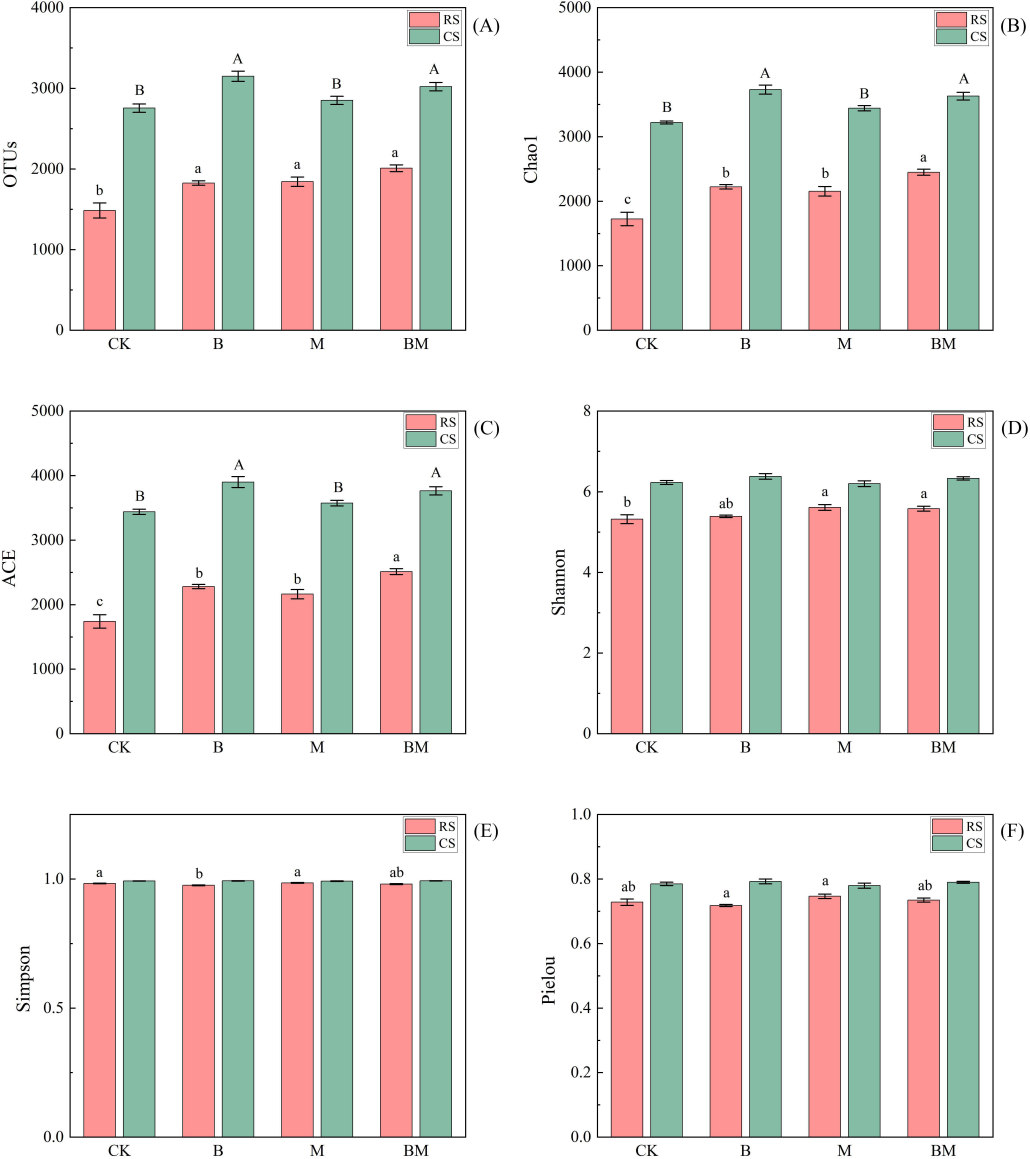
Effect of mowing and biochar on soil bacterial communities OTUs **(A)**, Chao1 **(B)**, ACE **(C)**, Shannon **(D)**, Simpson **(E)** and Pielou **(F)** in red soil (RS) and calcareous soil (CS). Values represent mean ± SE (*n = 4*). Different uppercase letters indicate significant differences among treatments in calcareous soil, different lowercase letters indicate significant differences among treatments in red soil (*p*≤0.05).

**FIGURE 2:**
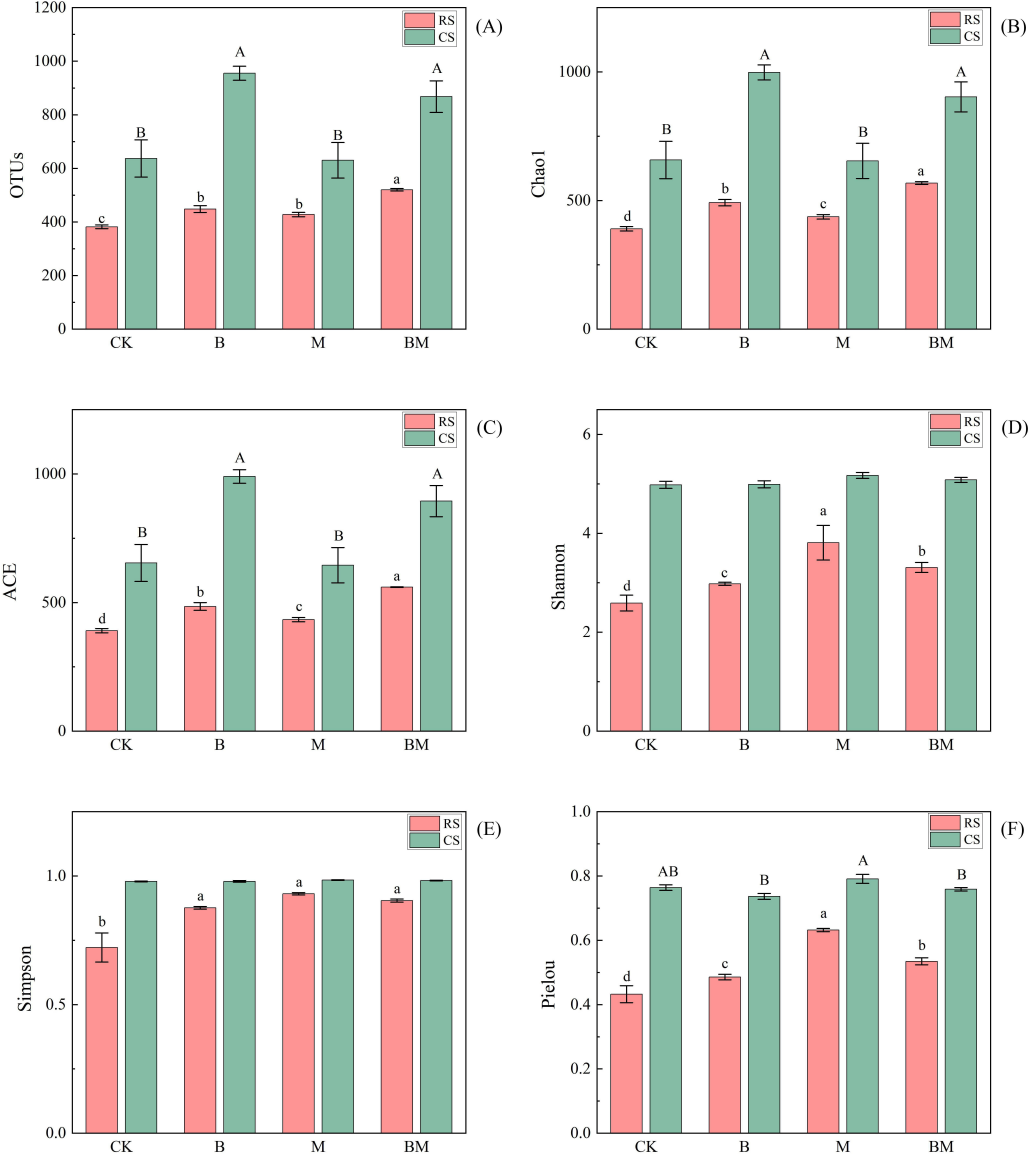
Effect of mowing and biochar on soil fungi communities OTUs **(A)**, Chao1 **(B)**, ACE **(C)**, Shannon **(D)**, Simpson **(E)** and Pielou **(F)** in red soil (RS) and calcareous soil (CS). Values represent mean ± SE (*n = 4*). Different uppercase letters indicate significant differences among treatments in calcareous soil, different lowercase letters indicate significant differences among treatments in red soil (*p*≤0.05).

In red soil, both biochar (B) and mowing (M), either alone or in combination (BM) treatments, significantly increased bacterial richness indices (OTUs, Chao1, ACE), with the greatest enhancement observed under the combined BM treatment. The M and BM treatments also significantly increased the Shannon index, while the Simpson index was significantly elevated by biochar alone. For fungal diversity, all indices–including OTUs, Chao1, ACE, Shannon, Simpson, and Pielou–were significantly increased by each treatment, with B, M, and BM all effective, and the BM treatment showing the strongest effect on richness (OTUs, Chao1, ACE). In contrast, responses in calcareous soil were more limited. Bacterial richness (OTUs, Chao1, ACE) increased significantly only under M and BM treatments, with no significant effect from B alone. For fungi, only the B and BM treatments significantly enhanced richness indices, while mowing alone had no significant impact (Figures 1, 2).

### 3.2 Microbial community structure and composition

**FIGURE 3:**
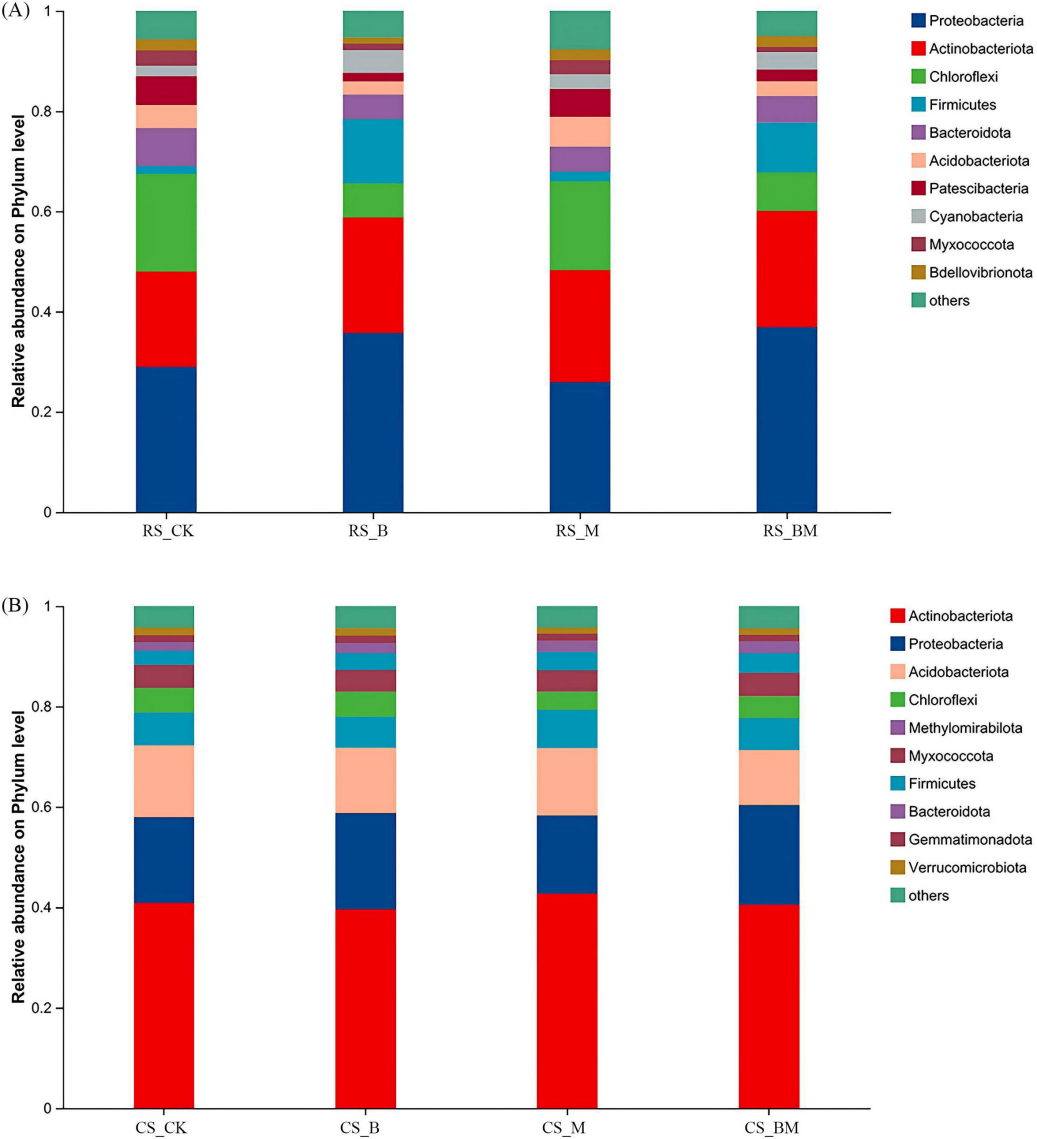
The relative abundance at the phylum level of bacterial communities. **(A)** Red soil **(B)** Calcareous soil. RS: Red soil; CS: Calcareous soil; CK: Control; B: Biochar treatment; M: Mowing treatment; BM: Biochar and Mowing treatment.

At the phylum level of bacterial communities in red soil, *Proteobacteria*, *Actinobacteria*, and *Chloroflexi* were the three most dominant taxa (Figure 3A). Following biochar application, the relative abundances of Proteobacteria and Actinobacteria were significantly increased, while that of *Chloroflexi* was notably reduced. In contrast, in calcareous soil, *Actinobacteria*, *Proteobacteria*, and *Acidobacteria* exhibited the highest relative abundances (Figure 3B), yet these phyla showed minimal shifts post-biochar treatment. Consequently, biochar exerted more pronounced effects on bacterial community structure in red soil compared to calcareous soil. Under mowing management, neither red nor calcareous soils displayed significant alterations in the relative abundances of dominant bacterial phyla, indicating structural stability. When biochar and mowing were combined, red soil exhibited a marked increase in *Proteobacteria* abundance alongside a significant decline in *Chloroflexi*. In calcareous soil, *Acidobacteria* abundance was moderately reduced under this combined treatment.

**FIGURE 4:**
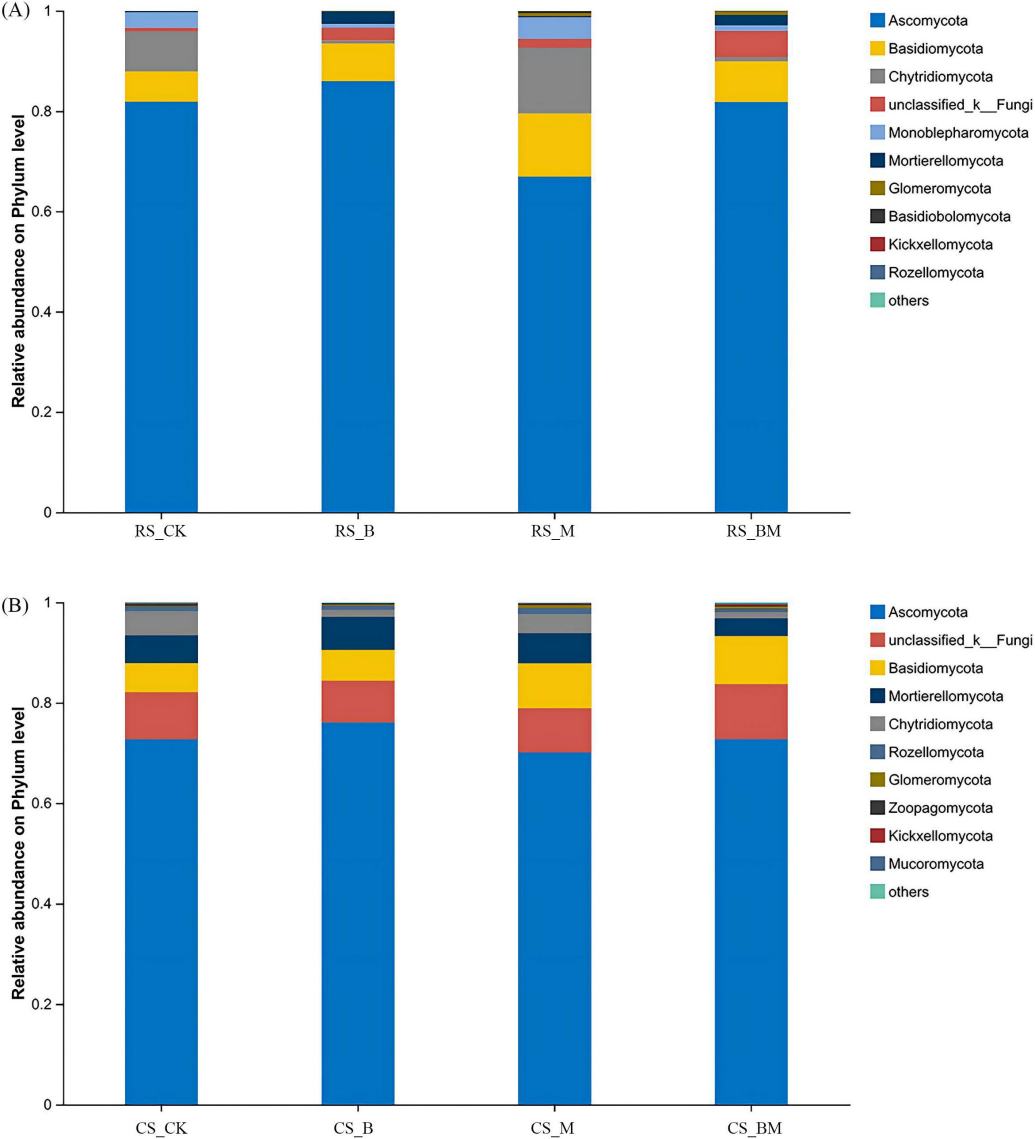
The relative abundance at the phylum level of fungal communities. **(A)** Red soil **(B)** Calcareous soil. RS: Red soil; CS: Calcareous soil; CK: Control; B: Biochar treatment; M: Mowing treatment; BM: Biochar and Mowing treatment.

In red soil fungal communities, *Ascomycota*, *Basidiomycota*, and *Chytridiomycota* dominated at the phylum level (Figure 4A). Biochar application significantly elevated the abundances of *Ascomycota* and *Basidiomycota* but reduced *Chytridiomycota*. Mowing, however, induced a contrasting response: *Ascomycota* decreased, while *Basidiomycota* and *Chytridiomycota* increased significantly. In calcareous soil, *Ascomycota*, unclassified_Fungi, and *Basidiomycota* were predominant (Figure 4B). These taxa remained largely unresponsive to biochar, underscoring the limited influence of biochar on fungal communities in calcareous soil compared to red soil. Mowing in calcareous soil triggered a modest but significant rise in *Basidiomycota*, though less pronounced than in red soil. Combined treatment in red soil reduced *Chytridiomycota* abundance, whereas calcareous soil saw increased *Basidiomycota* dominance.

### 3.3 Relationship between microbial community structure and soil physical and chemical properties

**FIGURE 5:**
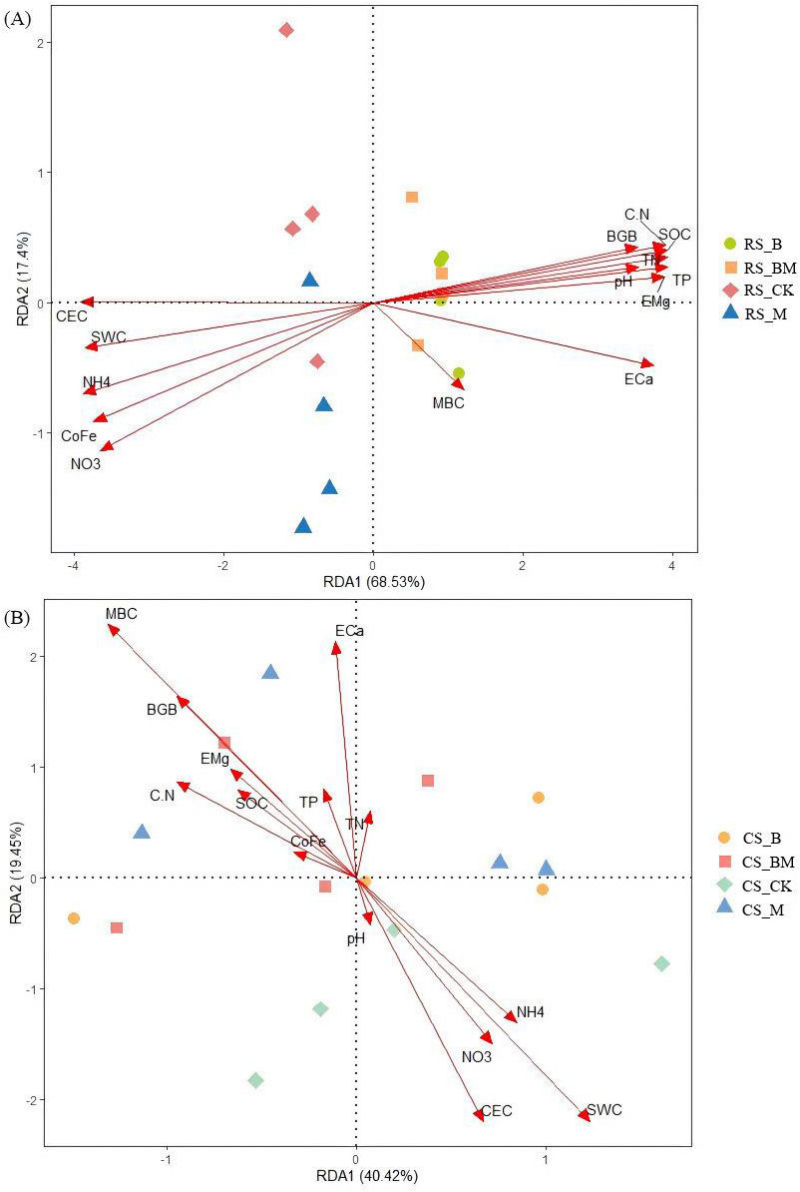
RDA analysis of the relationship between bacteria relative abundance at the phylum level and the soil physicochemical properties. **(A)** Red soil **(B)** Calcareous soil. RS: Red soil; CS: Calcareous soil; CK: Control; CK: Control; B: Biochar treatment; M: Mowing treatment; BM: Biochar and Mowing treatment; SWC: soil water content; SOC: soil organic carbon; TN: total nitrogen; TP: total phosphorus; C/N: C/N ratio; MBC: microbial biomass carbon; NH_4_^+^: ammonium nitrogen; NO_3_^–^: nitrate nitrogen; BGB: belowground biomass; CEC: cation exchange capacity; CoFe: complex iron; ECa: exchangeable calcium; EMg: exchangeable magnesium.

**FIGURE 6:**
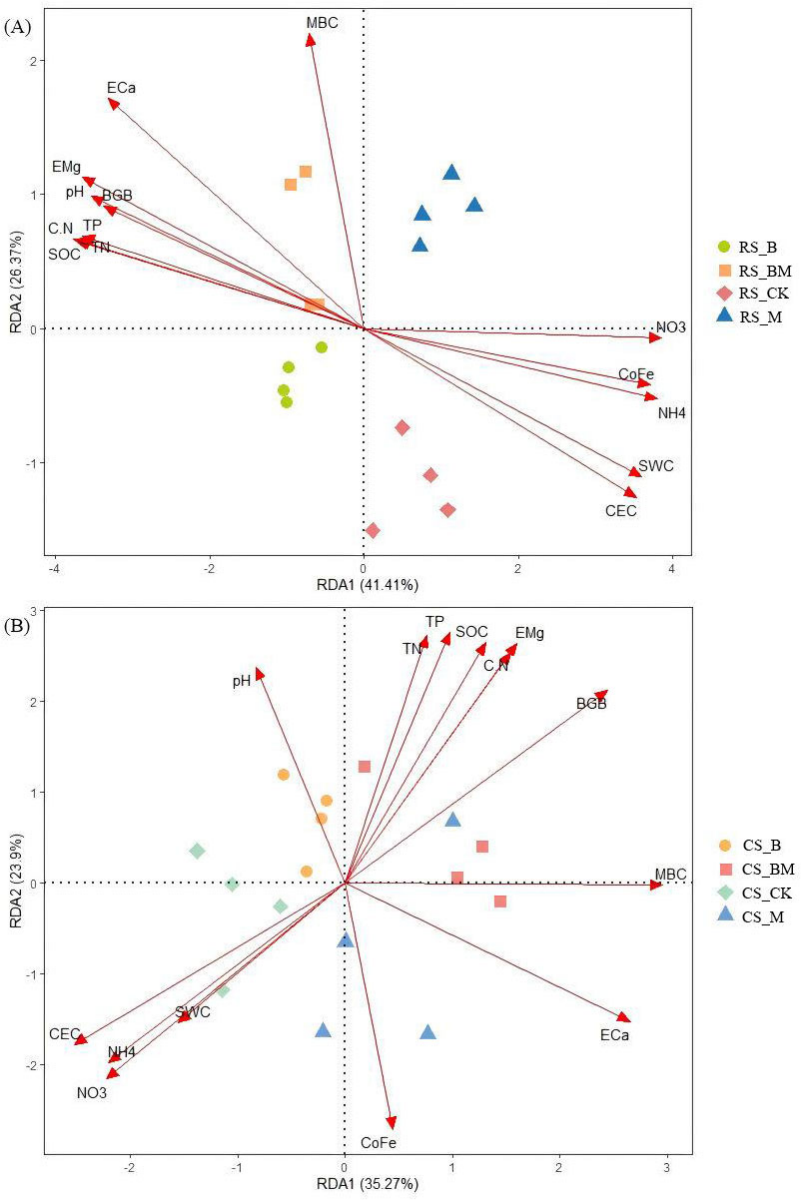
RDA analysis of the relationship between fungi relative abundance at the phylum level and the soil physicochemical properties. **(A)** Red soil **(B)** Calcareous soil. RS: Red soil; CS: Calcareous soil; CK: Control; B: Biochar treatment; M: Mowing treatment; BM: Biochar and Mowing treatment; SWC: soil water content; SOC: soil organic carbon; TN: total nitrogen; TP: total phosphorus; C/N: C/N ratio; MBC: microbial biomass carbon; NH_4_^+^: ammonium nitrogen; NO_3_^–^: nitrate nitrogen; BGB: belowground biomass; CEC: cation exchange capacity; CoFe: complex iron; ECa: exchangeable calcium; EMg: exchangeable magnesium.

Redundancy analysis (RDA) unveiled the covariation patterns between soil samples and environmental factors regulating bacterial and fungal community assembly, which significantly differed across soil types. The first two RDA axes explained 85.93% (Figure 5A) and 59.87% (Figure 5B) of the total variation in soil bacterial community structure. For fungal communities, the corresponding variations on the primary ordination axes were 67.78% (Figure 6A) and 59.17% (Figure 6B). This variance partitioning highlighted the differential dominance of environmental factors in microbial community assembly, with red soil exhibiting a stronger environmental filtering effect than calcareous soil.

**FIGURE 7:**
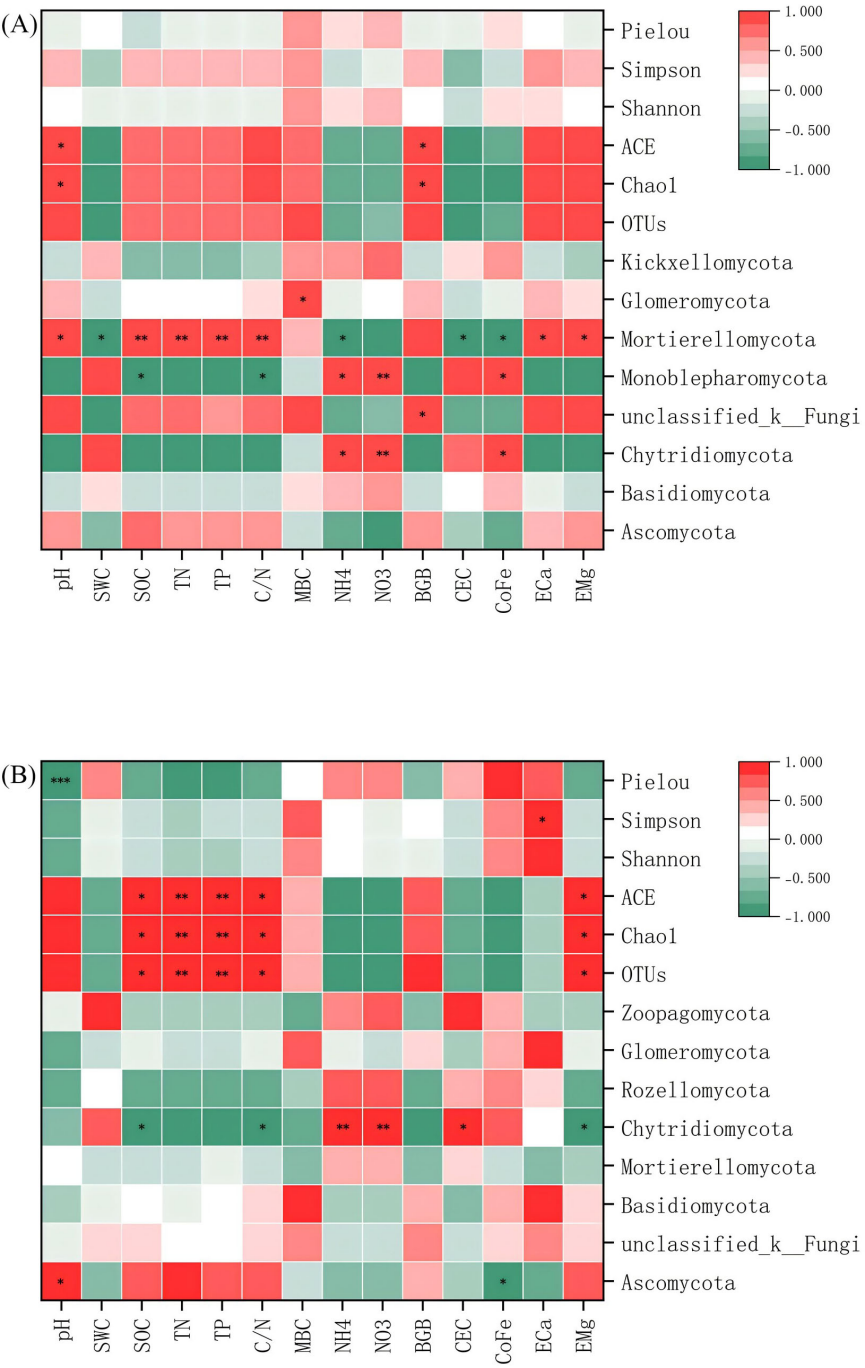
Pearson correlation heat map of bacterial communities, α -diversity and environmental factors at the phylum level. **(A)** Red soil **(B)** Calcareous soil. “*”, “**”and “***” are statistically significant at *P* < 0.05 and *P* < 0.01 and *P* < 0.001, respectively. SWC: soil water content; SOC: soil organic carbon; TN: total nitrogen; TP: total phosphorus; C/N: C/N ratio; MBC: microbial biomass carbon; NH_4_^+^: ammonium nitrogen; NO_3_^–^: nitrate nitrogen; BGB, belowground biomass; CEC, cation exchange capacity; CoFe, complex iron; ECa, exchangeable calcium; EMg, exchangeable magnesium.

**FIGURE 8:**
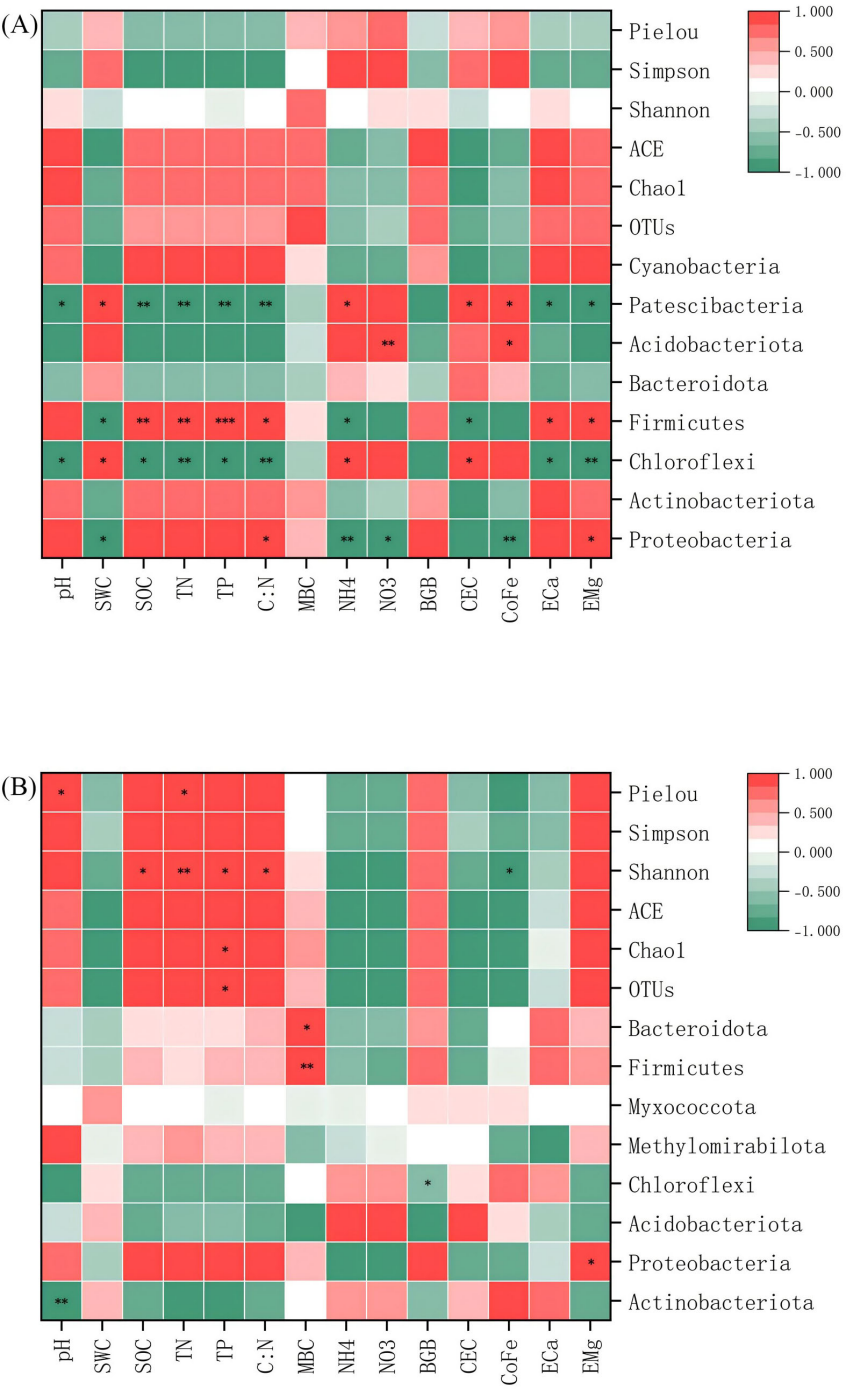
Pearson correlation heat map of fungi communities, α -diversity and environmental factors at the phylum level. **(A)** Red soil **(B)** Calcareous soil. “*”, “**”and “***” are statistically significant at *P* < 0.05 and *P* < 0.01 and *P* < 0.001, respectively. SWC, soil water content; SOC, soil organic carbon; TN, total nitrogen; TP, total phosphorus; C/N, C/N ratio; MBC, microbial biomass carbon; NH_4_^+^, ammonium nitrogen; NO_3_^–^, nitrate nitrogen; BGB, belowground biomass; CEC, cation exchange capacity; CoFe, complex iron; ECa, exchangeable calcium; EMg, exchangeable magnesium.

Pearson correlation analysis delineated statistically significant, specific associations between microbial communities, α diversity indices, and key environmental factors, uncovering niche differentiation patterns among microbial lineages. The correlations between microbial communities and environmental factors were more pronounced in red soil (*p* < 0.05). In bacterial communities, *Proteobacteria*, *Chloroflexi*, and *Firmicutes* showed significant positive and negative correlations with most environmental factors. Notably, *Chloroflexi* and *Firmicutes* exhibited opposite correlation patterns (Figure 7A). Within fungal communities, *Mortierellomycota* and *Monoblepharomycota* demonstrated significant correlations with most environmental factors (Figure 8A). Regarding α diversity, the correlations with environmental factors were more significant in calcareous soil (*p* < 0.05). The Shannon index of bacteria was significantly positively correlated with SOC, TN, TP, and C/N (Figure 7B). For fungi, ACE, Chao1, and OTUs exhibited significant positive correlations with SOC, TN, TP, and C/N (Figure 8B).

### 3.4 Physical and chemical properties of soil

Compared to the control (CK), biochar application significantly increased soil pH, organic carbon (SOC), total nitrogen (TN), total phosphorus (TP), C/N ratio, grass biomass (BGB), electrical conductivity (ECa), and exchangeable Mg (EMg) in red soil, while decreasing soil water content (SWC), NH_4_^+^-N, NO_3_^–^-N, cation exchange capacity (CEC), and Co-Fe content (CoFe). In calcareous soil, biochar induced similar changes across most variables, except that ECa remained unchanged. Mowing alone increased NH_4_^+^-N, NO_3_^–^-N, CoFe, ECa, and EMg in red soil, but reduced SWC and CEC. In contrast, in calcareous soil, mowing increased microbial biomass carbon (MBC), BGB, and ECa, while decreasing pH, NO_3_^–^-N, and CEC.

Under the combined biochar and mowing (BM) treatment, red soil showed significant increases in pH, SOC, TN, TP, C/N, MBC, BGB, ECa, and EMg, accompanied by decreases in SWC, NH_4_^+^-N, NO_3_^–^-N, CEC, and CoFe. In calcareous soil, the BM treatment elicited a largely similar response pattern, with the exception that pH and SWC did not differ significantly from the control (Supplementary Figure 1).

## 4 Discussion

### 4.1 The interactive effect of biochar and soil types on microbial diversity

The addition of biochar consistently enhanced microbial abundance and richness across both red and calcareous soils, corroborating numerous previous studies (Wang M. et al., 2023; Figures 1, 2). This effect is widely attributed to biochar’s porous structure and abundant surface functional groups, which provide physical refugia and nutrient sources for microorganisms, thereby promoting their colonization and proliferation (Barnes et al., 2014). Moreover, biochar improves soil physicochemical conditions by neutralizing acidic pH, enhancing soil aggregation, increasing total and capillary porosity, and improving aeration–collectively alleviating abiotic stresses such as drought and oxygen limitation (Murtaza et al., 2023). These modifications create heterogeneous microhabitats that support both aerobic and anaerobic microbial niches, further contributing to increased microbial activity and diversity (Lehmann et al., 2011). However, our study reveals a critical soil-type dependency in biochar’s impact on fungal diversity: significant increases in Shannon and evenness indices were observed only in acidic red soil (initial pH 5.28), not in neutral–alkaline calcareous soil (initial pH 7.96). This divergence highlights that the magnitude and direction of biochar-induced microbial responses are strongly mediated by pre-existing soil conditions.

In red soil, biochar raised the pH from 5.28 to 6.33, effectively alleviating acid stress–a known constraint on fungal growth (Rousk et al., 2010). This shift likely stimulated extracellular enzyme production, particularly those involved in carbon degradation (Luo et al., 2025), and favored the proliferation of decomposer taxa such as *Basidiomycota* (Tarin et al., 2021). In contrast, calcareous soil exhibited only a marginal pH increase (from 7.96 to 8.13), remaining within a range already favorable for most fungi. Consequently, the microbial community maintained its structural stability, with no significant change in diversity indices–consistent with reports that fungal communities in neutral–alkaline soils are less responsive to pH-modifying amendments (Xu et al., 2014). Notably, this pattern aligns with the “stress alleviation” hypothesis: biochar exerts the greatest positive effect in soils where key environmental constraints (e.g., acidity, nutrient deficiency) are present (Mansoor et al., 2021). In red soil, both pH correction and nutrient enrichment acted synergistically to relieve microbial stress. Conversely, in calcareous soil, the absence of strong limiting factors–combined with high inherent calcium content–may have dampened biochar’s influence. Indeed, calcium ions can bind with organic matter and biochar to form stable micro-aggregates (Jin et al., 2024), potentially reducing organic matter decomposition and slowing carbon release. This stabilization may reduce metabolic stress on microorganisms but also limit resource availability for fast-growing taxa, thereby maintaining community homogeneity.

Supporting this interpretation, biochar amendment in red soil led to substantial increases in soil organic carbon (SOC; from 3.16 to 98.05 g kg^−1^) and total nitrogen (TN; from 0.43 to 2.42 g kg^−1^), elevating the C:N ratio (Supplementary Figure 1). These changes alleviated nutrient limitations and favored copiotrophic microbial groups (e.g., *Proteobacteria, Actinobacteria*), while suppressing oligotrophic taxa (e.g., *Chloroflexi*)–a classic signature of resource-enriched environments (Fierer et al., 2012; Borny et al., 2024). Similarly, total phosphorus (TP) increased from 0.33 to 1.07 g kg^−1^, enhancing phosphorus availability and reducing microbial competition for this essential nutrient (Vance et al., 2003). Such nutrient enrichment likely promoted functional diversification and optimized community structure, particularly among fungi adapted to nutrient-rich conditions. In contrast, calcareous soil already possesses relatively high nutrient availability and pH buffering capacity, which may explain the muted microbial response. Furthermore, the formation of Ca–organic–biochar complexes may further stabilize carbon inputs, delaying nutrient mineralization and constraining the proliferation of opportunistic taxa (Abdul et al., 2021). This mechanism may contribute to the observed community stability rather than enhancement in diversity.

Therefore, our findings underscore that biochar does not universally enhance microbial diversity; its efficacy is contingent upon the initial soil environment. In degraded, acidic red soils–common in karst regions–biochar acts as a powerful restorative agent through dual pathways: pH amelioration and nutrient enrichment. In contrast, in already fertile and buffered calcareous soils, biochar’s effects are constrained by minimal environmental change and inherent biogeochemical stability. This context-dependent response reinforces the need for site-specific biochar management strategies rather than blanket application protocols. Our results extend previous work by demonstrating that fungal communities exhibit greater sensitivity to soil-type–mediated changes than bacterial communities (Figure 3), particularly in response to pH shifts. While bacteria are often more resilient to pH fluctuations due to shorter generation times and broader metabolic flexibility (Wang C. et al., 2023), fungi may serve as more sensitive bioindicators of soil restoration progress in red soil systems.

### 4.2 The interactive effect of mowing and soil types on microbial diversity

Mowing significantly influences soil microbial communities by altering plant residue decomposition dynamics and the quantity and quality of organic matter inputs (Pei et al., 2021). However, our results reveal that these effects are not uniform but are strongly modulated by soil type, highlighting a critical interactive effect between management practice and edaphic context (Figures 1, 2). In acidic red soils, mowing acts as a restorative disturbance, enhancing microbial diversity through multiple synergistic mechanisms. First, mowing stimulates root turnover and exudation (Luo et al., 2021), increasing the input of labile carbon substrates–such as sugars, organic acids, and amino compounds–that serve as readily available energy sources for microbes. This sustained carbon pulse helps alleviate the chronic carbon limitation typical of red soils (Johnson, 1998), thereby promoting microbial growth and species emergence. Second, mowing induces a slight but significant increase in soil pH, partially neutralizing the acidic environment. This pH shift reduces physiological stress on acid-sensitive taxa and activates pH-responsive bacterial groups (Wang et al., 2019), further contributing to community restructuring. Importantly, red soils typically exhibit low initial microbial diversity due to inherent acidity, low nutrient availability, and poor aggregation. This “low-diversity baseline” creates ecological opportunity–microbial communities in such stressed environments are more responsive to resource additions and environmental amelioration (Huston, 2014). Consequently, even modest improvements in carbon supply and pH can trigger substantial increases in diversity, as observed in our study (Liu et al., 2023). Thus, mowing in red soils functions as a facilitative perturbation, shifting the system toward a less stressed, more diverse state.

In contrast, calcareous soils respond to mowing differently–often negatively. These soils have high pH buffering capacity and abundant calcium, which naturally suppress organic matter mineralization due to carbonate stabilization (Gan et al., 2020). Mowing reduces aboveground biomass and photosynthetic return, leading to decreased labile carbon inputs and exacerbating resource scarcity. This constrains nutrient cycling and promotes carbon depletion. Calcareous soils typically have high initial microbial diversity, dominated by copiotrophic taxa adapted to fluctuating resources (Bossolani et al., 2021). Under mowing-induced carbon limitation, competition intensifies, favoring fast-growing specialists–such as *Actinobacteria*–over slower-growing or sensitive taxa (Hayat et al., 2021). Their high metabolic activity and antibiotic production further accelerate carbon turnover and suppress competitors, reducing evenness and functional redundancy. As a result, microbial communities become more homogeneous, limiting diversity gains. Additionally, mowing exerts minimal influence on soil pH in calcareous systems due to their strong buffering capacity. Without significant environmental amelioration, the potential for mowing to stimulate new microbial niches is limited. Thus, rather than acting as a facilitator, mowing in calcareous soils functions as a competitive stressor, reinforcing existing dominance patterns and constraining diversity gains.

Overall, our findings demonstrate a dichotomous response to mowing: in red soils, mowing alleviates abiotic stress (acidity) and biotic constraints (carbon limitation), thereby enhancing microbial diversity–a pattern consistent with the “stress-gradient hypothesis” (Daebeler et al., 2020; Maestre et al., 2006), where facilitative interactions dominate under harsh conditions. Conversely, in calcareous soils, mowing intensifies resource competition in an already competitive environment, yielding neutral or negative outcomes for diversity. This contrast emphasizes that the same management practice can elicit opposite ecological responses depending on the initial soil context. These results have important implications for sustainable grassland and agroecosystem management. They suggest that mowing may be most beneficial in degraded, acidic soils, where it can promote microbial recovery, but should be applied cautiously in calcareous systems, where it may disrupt established microbial networks and reduce functional resilience.

### 4.3 The potential synergistic trend of the combined processing

The potential synergistic effect of biochar amendment and mowing on microbial biomass and diversity is more evident in red soil than in calcareous soil, reflecting fundamental differences in soil geochemistry and microbial constraints (Figures 1, 2). In red soil, long-term biochar application alleviates key limitations: it neutralizes soil acidity, provides stable carbon substrates, improves pore structure, and enhances habitat connectivity for microbes (El-Sharkawy et al., 2022). When combined with mowing–which stimulates root exudation and litter inputs–this creates a dual carbon supply: recalcitrant biochar-derived carbon sustains slow-growing, stress-tolerant taxa, while labile plant-derived carbon fuels copiotrophic populations (Wu et al., 2018). This complementary resource input reduces nutrient competition and promotes both bacterial and fungal growth, suggesting a synergistic interaction that enhances overall microbial biomass and diversity. In contrast, such synergy is constrained in calcareous soils. The inherently high pH and carbonate buffering capacity limit biochar’s ability to further modulate soil chemistry, while Ca^2 +^ saturation inhibits the oxidative degradation of biochar surfaces, reducing its bioavailability and slow-release carbon function (Rahmanian and Khadem, 2024). Moreover, calcium ions readily form organo-mineral complexes with mowing-derived organic residues, decreasing substrate accessibility for decomposers and suppressing microbial activity. Mowing alone has minimal impact on microbial diversity in this context, indicating low responsiveness to disturbance. Consequently, the ecological opportunity for synergy is greater in red soils–where abiotic stress and carbon limitation create a “responsive” microbial community–than in calcareous soils, where chemical stability and pre-existing resource constraints dampen the effects of both interventions. Thus, the expression of synergistic trends depends not only on the combination of management practices but critically on the underlying soil context.

In addition to above, our results also demonstrate a significant synergistic effect between biochar application and mowing (BM), which markedly enhanced aboveground biomass in both red soil (RS) and calcareous soil (CS) (Supplementary Figure 1A). This synergy likely arises from biochar-improved soil water retention and nutrient-holding capacity, which facilitate rapid plant regrowth following mowing, while returned plant residues promote organic matter accumulation and microbial activity, accelerating nutrient mineralization and establishing a “soil-plant-management” positive feedback loop (Pan et al., 2025; Hao et al., 2025). This synergistic effect represents a key practical outcome of our research, highlighting a promising integrated management strategy for restoring productivity in degraded grasslands. Surprisingly, biochar application (B and BM) significantly reduced soil cation exchange capacity (CEC) (Supplementary Figure 1L), contrary to the widely reported CEC-enhancing effect (Pidlisnyuk et al., 2025). This anomaly may be attributed to the high-temperature production (>500 °C) of the biochar used, which promotes extensive aromatization and thermal degradation of surface functional groups, thereby reducing negative charge density (Luo et al., 2023). Additionally, abundant Ca^2+^ and Mg^2+^ in biochar ash (as shown in Supplementary Figures 1N, O, where B and BM treatments increased calcium and magnesium ion concentrations) may induce “charge shielding” or “ion bridging” effects (Zhang et al., 2025), while biochar-iron/aluminum oxide interactions via ligand exchange or surface complexation could mask variable-charge surfaces (Yuan et al., 2025). Biochar-derived dissolved organic matter (DOM) may also neutralize clay surface charges under certain conditions, further reducing effective CEC (Li et al., 2025). In variable-charge soils, pH elevation from biochar may alter surface charge dynamics near the point of zero charge (PZC), and concurrent base cation inputs could lead to ion competition or precipitation (e.g., calcium carbonate), affecting CEC measurements (Fung et al., 2023).Furthermore, the significant reduction in plant-available nitrogen (NH_4_^+^ and NO_3_^–^) in biochar-amended pots (Supplementary Figures 1 J, K) is a crucial finding for nutrient management. We postulate that biochar-induced carbon surplus disrupted the soil C:N balance, triggering rapid microbial growth and short-term nitrogen immobilization or “lock-up” (Muhammad et al., 2023). This effect was amplified in the BM treatment due to the co-addition of labile carbon from residues and stable carbon from biochar, creating a synergistic “activation effect” that intensified microbial nitrogen competition (Chamoli et al., 2025). Consequently, early plant growth in nutrient-poor soils may be temporarily limited, necessitating careful nitrogen fertilization. However, in the long term, this mechanism promotes a “slow-release” nitrogen supply by reducing nitrate leaching and N_2_O emissions, thereby improving nitrogen use efficiency through gradual mineralization. Biochar thus functions not merely as a passive adsorbent but as an active regulator of soil nitrogen dynamics, with important implications for sustainable soil management.

### 4.4 Correlations between microbial diversity, community structure and soil properties

Microbial community diversity is significantly correlated with soil physicochemical properties (Figures 7, 8), indicating that edaphic factors play a central role in shaping microbial community structure and function. However, the nature and strength of these relationships differ markedly between red and calcareous soils, reflecting divergent ecological constraints.

In red soils, microbial community structure is strongly driven by pH, soil organic carbon (SOC), total nitrogen (TN), total phosphorus (TP), and C/N ratio. The typically low pH of red soils imposes abiotic stress, directly inhibiting microbial enzyme activity and compromising membrane integrity (Wang et al., 2024), while also selecting for acid-tolerant taxa. Svenningsen et al. (2018) have shown that pH is a master driver of microbial biogeography, and even small shifts can restructure communities. Concurrently, low SOC and nutrient availability create strong carbon and nutrient limitation, making microbial communities highly responsive to organic inputs (Goldfarb et al., 2011). This sensitivity is evident in our results: biochar addition increased the relative abundance of *Proteobacteria*–a typically copiotrophic phylum capable of rapid growth under resource-rich conditions–while decreasing *Chloroflexi*, which are often oligotrophic and adapted to low-energy environments (Arunrat et al., 2022; Figure 3). Similarly, *Firmicutes* showed significant positive correlations with SOC, TN, and TP in red soils, consistent with their role in organic matter decomposition and fermentation (Bonanomi et al., 2016), whereas *Chloroflexi* were negatively correlated, reflecting their competitive disadvantage under elevated nutrient availability.

In contrast, microbial α-diversity in calcareous soils is more strongly influenced by phosphorus dynamics and pH stability. The high calcium content promotes the formation of insoluble calcium-phosphate complexes, limiting phosphorus bioavailability and intensifying competition among microbes–particularly between phosphate-solubilizing bacteria and generalists (Vyas and Gulati, 2009). This resource competition becomes a key regulator of diversity. Meanwhile, the inherent carbonate buffering system maintains a stable, near-neutral to alkaline pH, which falls within the optimal range for many microorganisms. This stability reduces environmental filtering and community turnover, thereby supporting higher baseline α-diversity (Fan et al., 2024). Unlike in red soils, correlations between major microbial taxa (e.g., *Firmicutes*, *Chloroflexi*) and nutrient indices were not significant in calcareous soils, suggesting a decoupling of community composition from short-term nutrient fluctuations.

Overall, these contrasting patterns highlight that soil type fundamentally shapes microbial responses: in red soils, abiotic stress (acidity) and resource scarcity (C, N, P limitation) make microbial communities highly sensitive to management-induced changes, with community structure tightly linked to nutrient availability. In calcareous soils, chemical stability and P limitation dominate, shifting the primary response to competition-driven dynamics and stabilizing α-diversity. Thus, the regulation of microbial communities is not solely a function of individual soil properties, but of the integrated physicochemical context that defines stress thresholds and resource strategies.

### 4.5 Limitations and future Directions

Based on our results and supporting literature (Xiao et al., 2016; Cen et al., 2021; Chen et al., 2021; Guo et al., 2025), we now propose the following field-applicable recommendations. The biochar application rate in this experiment was 10% (w/w), equivalent to approximately 240 t/ha assuming a 20-cm plow layer and a soil bulk density of 1.2 t/mł. While this is higher than typical field applications (usually 5–50 t/ha), it was chosen to clearly detect microbial responses under controlled conditions. Future field studies should test lower, more practical rates (e.g., 20–30 t/ha) to evaluate scalability. Therefore, our findings offer practical insights for restoring degraded karst grasslands. In red soils, where both biochar and mowing significantly enhanced microbial communities, a combined management strategy is recommended: applying biochar at a rate of 20–30 t/ha (e.g., Xiao et al., 2016; Cen et al., 2021), which are economically and logistically feasible while still likely to enhance microbial communities, especially in red soils, and followed by moderate mowing every 6–8 weeks during the growing season, avoiding over-harvesting. This balances biomass removal with plant recovery and root exudation, which likely supports microbial growth. This regime may promote root exudation and organic input while maintaining plant productivity, potentially synergizing with biochar to boost microbial activity. In calcareous soils, where mowing had minimal effects, biochar application alone (at the same rate) may be sufficient to improve microbial conditions. Given the high cost and labor of mowing, this suggests a more targeted, soil-specific approach to restoration. Future field trials should validate these recommendations under real-world conditions, particularly regarding long-term carbon sequestration and plant community recovery.

Although the experiment was carefully controlled, several potential sources of error should be acknowledged. First, despite using a randomized block design and weekly pot rotation, subtle microclimatic gradients–such as variations in light intensity and temperature–within the greenhouse may have introduced residual variability. Second, although soil and plant sampling procedures were standardized, manual collection could lead to minor positional differences within pots. Third, technical variation inherent in high-throughput sequencing and enzyme assays may have influenced the precision of microbial measurements. Nevertheless, the consistency of treatment effects across replicates and the statistical significance of key interactions indicate that these potential errors did not compromise the main conclusions.

Several additional limitations also warrant consideration. First, the study was conducted under controlled mesocosm conditions with a limited number of replicates (*n* = 4). While sufficient to detect strong treatment effects, higher replication in future field studies would enhance statistical power and better account for micro-scale environmental heterogeneity. Second, mesocosm systems necessarily simplify natural ecosystems. Therefore, our findings should be validated through field-scale restoration experiments in actual degraded karst grasslands, where abiotic and biotic interactions are more complex and dynamic. Finally, the experiment spanned one year; given that biochar effects can evolve over time and microbial communities may undergo succession, long-term monitoring (e.g., 3–5 years) is recommended to evaluate the stability and sustainability of the observed responses. Such studies will be essential for developing effective, science-based restoration strategies for karst ecosystems.

## 5 Conclusion

This study reveals that the parent soil type is a critical determinant of soil microbial responses to biochar application and mowing–a key insight with broad implications for sustainable soil management in heterogeneous landscapes. While biochar consistently enhanced microbial abundance and richness across both red and calcareous soils, its effects on fungal diversity and community structure were pronounced only in red soil. Similarly, mowing significantly boosted microbial metrics in red soil but had no detectable impact in calcareous soil, underscoring the soil-specific nature of management outcomes. Notably, the combined biochar–mowing (BM) treatment in red soil showed a potential synergistic effect, suggesting that integrated practices may amplify microbial recovery in degraded ecosystems. Stronger correlations between soil physicochemical properties and microbial communities in red soil further indicate a more responsive and manageable microbiome in this substrate. These findings highlight that one-size-fits-all management strategies are unlikely to succeed in regions with diverse soil types, such as karst ecosystems. Instead, interventions should be tailored to local soil conditions. We recommend that future research validate these results through well-replicated field trials, long-term monitoring, and expanded experimental replication. Such studies will be essential to confirm the durability of microbial responses, improve statistical power, and elucidate the mechanisms behind observed interactions under real-world conditions. In the meantime, our results support the targeted use of biochar combined with moderate mowing as a promising strategy for enhancing soil health in red soil systems–with adjustments based on site-specific conditions and validated through robust experimental design.

## Data Availability

The raw sequencing data generated in this study have been deposited in the NCBI Sequence Read Archive (SRA) under BioProject accession number (PRJNA1338870). The data are publicly available at: https://www.ncbi.nlm.nih.gov.
