# Supplementary material for: Parent soil type modulates biochar and mowing effects on soil microbial communities in karst region
Source: Front Microbiol. 2025 Oct 28;16:1680847. doi: 10.3389/fmicb.2025.1680847 (PMC12602502; doi:10.3389/fmicb.2025.1680847)

Supplementary Material

# Supplementary Tables

**Table S1.** Initial physicochemical properties of two soil types.The results were given as Mean ± SE (Standard Error). Red soils (RS); Calcareous soils (CS); soil organic carbon (SOC); soil total nitrogen (TN); soil total phosphorus (TP); soil microbial biomass carbon (MBC).

| Soil Type | pH | SOC(g/kg) | TN(g/kg) | TP(g/kg) | MBC(mg/kg) |
| --- | --- | --- | --- | --- | --- |
| RS | 5.28±0.03 | 3.16±0.22 | 0.43±0.02 | 0.33±0.01 | 37.55±1.94 |
| CS | 7.78±0.02 | 23.61±0.76 | 2.16±0.03 | 0.72±0.03 | 336.52±0.76 |

**Table S2.** The key physicochemical properties (e.g., pH, organic carbon (OC), total nitrogen (TN), total phosphorus (TP), total Potassium (TK), Hydrogen, Ash content, Conductivity, Specific surface area and Average pore size) of the biochar used.

| **Metric** | **Value** |
| --- | --- |
| OC(%) | 42.08 |
| TN(%) | 1.49 |
| TP(%) | 2.36 |
| TK(%) | 15.82 |
| Hydrogen(%) | 1.08 |
| pH | 9.46 |
| Ash content(%) | 8.43 |
| Conductivity(mS/cm) | 1.22 |
| Specific surface area(m²/g) | 68.00 |
| Average pore size(nm) | 7.45 |

# Supplementary Figures

**Figure S1.** Effect of mowing and biochar on soil **(A)** Aboveground biomass (AGB), **(B)** Belowground biomass (BGB), **(C)** pH, **(D)** soil water content (SWC), **(E)** organic carbon (SOC), **(F)** total nitrogen (TN), **(G)** total phosphorus (TP), **(H)** C/N ratio (C/N), **(I)** microbial biomass carbon (MBC), **(J)** ammonium nitrogen (NH_4_^+^), **(K)** nitrate nitrogen (NO_3_^-^), **(L)** cation exchange capacity (CEC), **(M)** complex iron (CoFe), **(N)** exchangeable calcium (ECa), **(O)** exchangeable magnesium (EMg) in red soil (RS) and calcareous soil (CS). Values represent mean ± SE (n = 4). Different uppercase letters indicate significant differences among treatments in calcareous soil, different lowercase letters indicate significant differences among treatments in red soil (p ≤ 0.05).


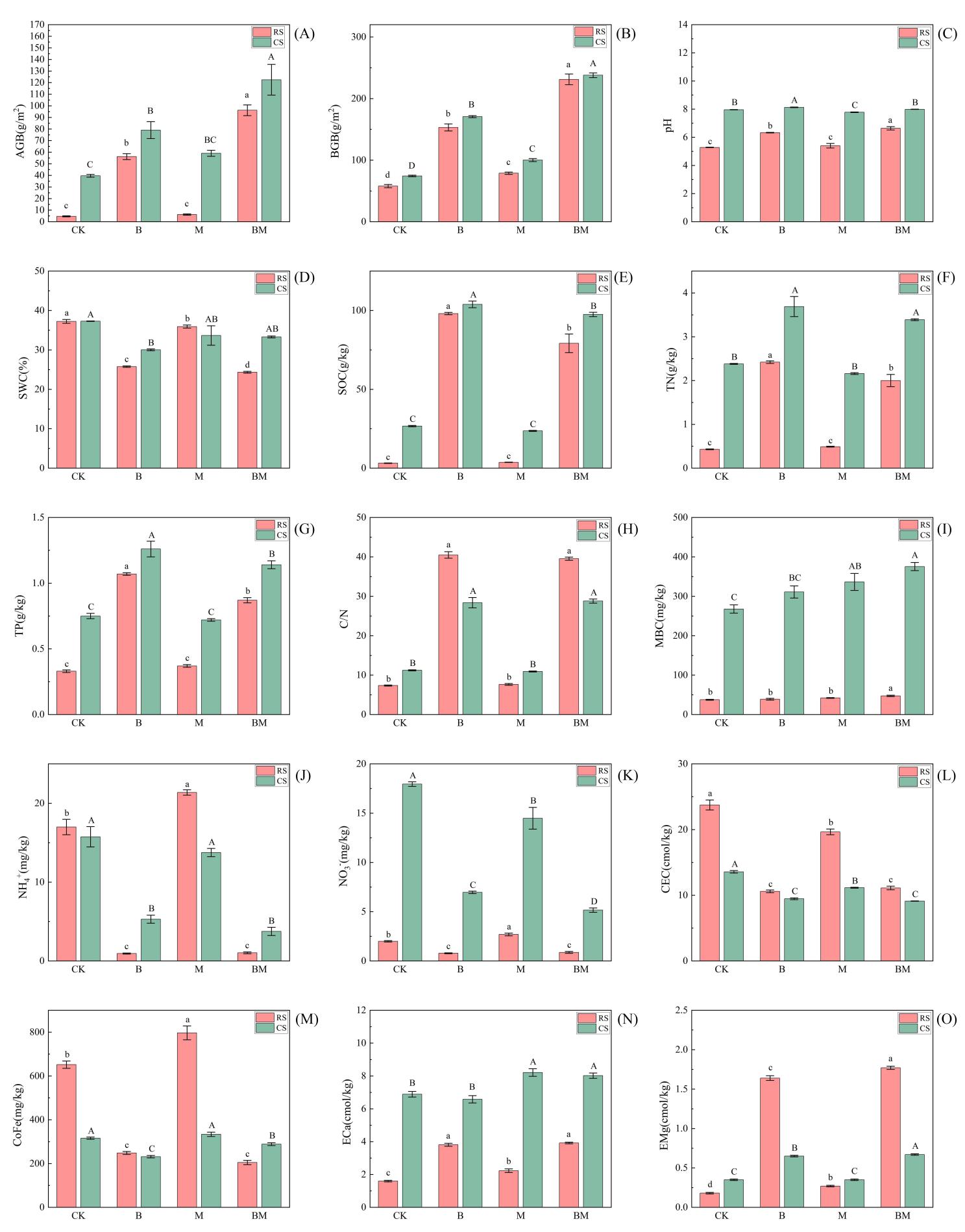

Supplement: Supplementary file 1 [file Data_Sheet_1.docx]
